# From rote to reasoned: Examining the role of pedagogical reasoning in practice-based teacher education

**DOI:** 10.1016/j.tate.2019.102991

**Published:** 2020-03

**Authors:** Sarah Schneider Kavanagh, Jenni Conrad, Sarah Dagogo-Jack

**Affiliations:** a University of Pennsylvania, USA; b University of Washington, USA; c Lawrence Public Schools, USA

## Abstract

• Critics worry that practice-based teacher education focuses on teaching behaviors and not pedagogical reasoning.
• Practice-based teacher learning experiences can be aimed at developing pedagogical reasoning.
• Teacher educators use particular practices to highlight pedagogical reasoning when working on practice with novices.
• We need more nuanced frameworks for describing, implementing, and studying teacher education pedagogy.

Ambitious teachers engage students in collaborative negotiations of ideas motivated by authentic questions (Moje, 2015; Shanahan & Shanahan, 2012; Windschitl, Thompson, Braaten, & Stroupe, 2012; Wineburg, Martin, & Monte-Sano, 2013). Such instruction requires teachers to be adept at using students’ ideas as fuel to power classroom activity (Nystrand, Wu, Gamoran, Zeiser, & Long, 2003). Enacting ambitious teaching is complex work because of the sophistication required to make quick decisions about how to meaningfully use emergent student ideas (Lampert, Beasley, Ghousseini, Kazemi, & Franke, 2010). The complexity of ambitious teaching raises important questions for teacher education. Namely, what kinds of experiences and guidance do novices need to become skilled in making rapid, reasoned, in-the-moment instructional decisions? Studies into the pedagogy of teacher education have begun to investigate questions like this by testing novel structures and activities for teacher learning, like studio-days (Von Esch & Kavanagh, 2018), rehearsals (Lampert et al., 2013), video clubs (van Es & Sherin, 2008), mediated fieldwork (Zeichner, 2010), and learning labs (Gibbons, Kazemi, & Lewis, 2017). This body of research has offered new tools for teacher educators (TEs) interested in developing teachers who can skillfully reason in practice.

TEs have benefited from this proliferation of activity structures for working on practice with teachers. However, there remains a paucity of frameworks for studying the pedagogy of TEs *while* enacting such activities. One notable exception is Grossman and her colleagues’ framework (Grossman, Compton, et al., 2009), which offers three constructs for studying teacher education pedagogy: representations, decompositions, and approximations of practice. These three constructs have become the backbone of research on the pedagogy of teacher education (Boerst, Sleep, Ball, & Bass, 2011; Ghousseini & Herbst, 2016; Grossman et al., 2019; Hauser & Kavanagh, 2019; Kavanagh & Danielson, 2019; Kavanagh, Monte-Sano et al., 2019; Reisman et al., 2019; Reisman et al., 2018; Wetzel, Rosner, Hoffman, Martinez, & Price-Dennis, 2016). While a close read of Grossman, Compton, et al.’s (2009) framework offers a sophisticated vision of teaching practice grounded in sociocultural theory, the three prongs of the framework are sometimes discussed by others without a clear perspective about the *object* of teacher learning in practice. In other words, when discussing the importance of representing practice, what is it that we, as a field, believe is important to represent? Is it what Kennedy (2016) would call the visible behaviors of teaching or is it the interplay between thought, action, identity, and knowledge? And if it is the latter, what does that look like?

Answering these questions is important because there are some emerging examples of teacher education that prioritize representations, decompositions, and approximations, but do so with an aim to develop teachers who enact moves before they have time to consider them. For example, some alternative teacher education programs in the United States have grounded their approaches to practicing teaching in popular texts such as Lemov, Woolway, and Yezzi’s (2012) *Practice perfect: 42 Rules for getting better at getting better,* in which rule number three is “Let the Mind Follow the Body” (p. 32). Practice opportunities that follow this rule are intentionally designed to support teachers to adopt actions without thinking about them. According to the authors*,* “once you have learned a skill to automaticity, your body executes, and only afterwards does your mind catch up … [you] do it without thinking and this is exactly the point. . . . The takeaway: You don’t have to be aware of your knowledge to use it. In fact, awareness often gets in the way” (pp. 33–36). Alternative teacher education programs that have adopted this Pavlovian perspective on teacher learning often represent, decompose, and approximate visible behaviors of teaching, such as “scanning the room,” “using non-verbal signals,” or “giving precise praise” in the hopes that these moves become routine behaviors that teachers execute without engaging in conscious thought (Lemov, 2012).

While TEs, programs, and graduate schools that have adopted this approach are certainly representing, decomposing, and approximating practice, their vision of *practice* seems to prioritize the visible behaviors of teaching over the underlying thoughts, knowledge, and beliefs that animate them (Kennedy, 2016). In a moment when the field is locked in debates about whether practice-based teacher education (PBTE) is professionalizing or de-professionalizing and humanizing or de-humanizing (Philip et al., 2019), researchers need conceptual tools that can support them in identifying approaches to representing, decomposing, and approximating teaching that prioritize pedagogical reasoning and discourage technocratic approaches to teaching. Towards this end, we studied the pedagogy of TEs within a program that described itself as “practice-based,” but was organized around practices such as “positioning students as competent sensemakers” and “orienting students to each other’s ideas” (for a full list, see Kavanagh & Rainey, 2017, p. 912). Our research was organized around the following question:

*When working with novice teachers on practice, to what extent and in what ways did teacher educators (TEs) mediate novices’ opportunities to engage in pedagogical reasoning?*

Our findings have implications for research on teacher education pedagogy as well as implications for TEs interested in developing their practice, especially those interested in adopting PBTE in ways that push against technocratic visions of teaching.

## 1 Practice-based teacher education pedagogy

Our research is motivated by an interest in understanding whether and how TEs supported novices to engage in pedagogical reasoning when they represented, decomposed, and approximated teaching practice. To introduce this investigation, we revisit Grossman and colleagues’ (Grossman, Compton, et al., 2009) framework, which presents three pedagogies used by professional educators to support professional learning about practice. The first, *offering representations of practice*, describes how professional educators support novices to envision professional practice. TEs offer representations of practice when they screen videos of teaching, model teaching, or examine cases or lesson plans. Representations vary in the extent to which they comprehensively and authentically illustrate the relationship between teachers’ actions and teachers’ thinking. An unannotated video of teaching, for example, offers no information about *why* a teacher took the actions captured in the footage. This variation likely influences the extent to which a given representation can support teachers in learning about the interplay of knowledge and action.

The second element of Grossman, Compton, et al.’s (2009) framework is *decompositions of practice*: breaking down the complex work of teaching into named parts. This breaking down enables novices to cultivate a nuanced professional vision and language: through categorization, novices gain a better understanding of what to look for and how to describe and interpret what they see. By identifying and naming the component parts of practice, TEs support novices to practice and use the language of their profession. The challenge remains that separating the part (a pedagogical move) from the whole (teaching practice writ large) can alternately illuminate and minimize the complex practice of experienced teachers. Opportunities for re-composition, or integrating these parsed skills and components into more authentic and complex practice, remain essential for novices to attain a broader and deeper professional vision and practice (Janssen, Grossman, & Westbroek, 2015).

The final element of Grossman, Compton, et al.’s (2009) framework is *approximations of practice*. Approximations simulate the most challenging aspects of the profession, helping novices try out their craft in safe waters. Through low-risk experiments in acting out the practice of teaching, and experiencing instructive failure, novice thinking can approach more specificity and depth. As with decompositions, effective approximations of practice rely on some inauthenticity: focusing on one difficult aspect of teaching (e.g., asking open-ended, text-dependent questions) necessarily overlooks the range and difficulty of skills needed to practice that aspect within the context a chaotic classroom.

Though Grossman, Compton, et al.’s (2009) framework is widely used, the field’s lexicon for describing teacher education pedagogy remains limited, particularly in verbs. We have myriad ways of describing the object of teacher learning (Darling-Hammond & Bransford, 2005) as well as its purpose (Cochran-Smith et al., 2012), but as a field, we have struggled to describe the *activity* of learning to teach (Lampert, 2010). While there is a large body of work on how to prompt and support teachers *to reflect* (Brandenburg, Glasswell, Jones, & Ryan, 2017; Gore, 1987; Gore & Zeichner, 1991; Schön, 1983; Sparkes, 1991) and *to inquire* (Bird & Little, 1986; Carr & Kemmis, 1986; Cochran-Smith & Lytle, 1993, 2009), researchers have few other verbs to draw from when describing the activity of learning to teach. By introducing “represent,” “decompose,” and “approximate” into the field’s lexicon for describing the activity of learning to teach, Grossman, Compton, et al.’s (2009) framework expanded the linguistic landscape of our ability to describe the activity of teacher education (see also McDonald, Kazemi, & Kavanagh, 2013; Lampert et al., 2013). However, the field still lacks a language for describing teacher education pedagogy at a finer grain. “Representations” offer us a broad category, but what are the characteristics of representations that support teachers to see the interplay between the visible and invisible parts of teaching? What is the difference between approximations that support teachers to attempt behaviors absent thought and approximations that support teachers to attempt embodied pedagogical reasoning? To draw a comparison to K-12 instruction, representation/decomposition/approximation is to PBTE what the writing process (prewriting/drafting/revising/publishing) is to writing instruction: It offers a unit-level structure that can be facilitated at vastly differing levels of sophistication by individual TEs.

Sophisticated representations, decompositions, and approximations link the activity of teaching with the knowledge and ethical infrastructure that animate it. When teacher education pedagogy focuses only on the visible elements of teaching practice (“what teachers do”), TEs can entirely overlook the invisible yet essential elements of pedagogical reasoning and professional judgment (“why they do it”). Teacher education that employs representation, decomposition, and approximation to model and teach ambitious instruction must take seriously this challenge. To design more precise approximations of practice, ambitious teacher education must involve unfolding the invisible professional thinking behind discrete elements of teaching practice; behaviors alone cannot constitute ambitious or effective teaching (Kennedy, 2016).

### 1.1 Pedagogical reasoning

The invisible cognitive work undergirding ambitious teaching is often called *pedagogical reasoning,* what Loughran (2019) described as “the thinking that underpins informed professional practice” (pp. 4). Pedagogical reasoning, also referred to as “instructional reasoning” (Tiilikainen, Toom, Lepola, & Husu, 2019), is the activity through which teachers attach their actions to the purposes that undergird them. Such reasoning is always situated in an instructional context because instructional purposes arise in response to particular dilemmas sitting at the nexus of teachers, students, content, and context (Chazan, Herbst, & Clark, 2016; Klette, 2007; Shulman, 1987; Stenberg, Karlsson, Pitkäniemi, & Maaranen, 2014; Tiilikainen et al., 2019; Zierer, 2015).

**Fig. 1:**
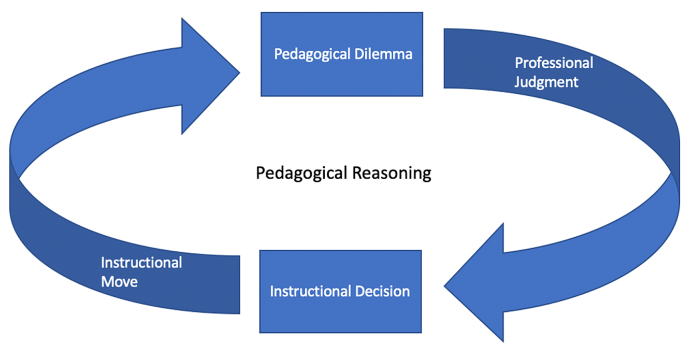
A Model of Pedagogical Reasoning.

Guided by scholarship on pedagogical reasoning, we see teaching as a dynamic interplay between knowing and doing (Dewey, 2010), in which teachers are decision-makers who consistently face pedagogical dilemmas and employ professional judgment. This perspective draws on the concept of the *pedagogical dilemma*, a phrase coined by Lampert (1985) to describe those moments when teachers face equally reasonable alternatives while teaching that require reasoned arguments with oneself. These dilemmas occur for teachers in a steady stream (e.g., who to call on, when to offer input, when to hold back). Through this lens, teaching is the act of consistently choosing between alternative courses of action, all of which will create new pedagogical dilemmas. To navigate this terrain, teachers rely on their professional judgment, their ability to make decisions in moments of uncertainty that are productive for student learning. Shulman (1998) described professional judgment as the negotiation of “the universal terms of theory and the gritty particularities of situated practice” (p. 519). In short, we see pedagogical reasoning as the act of drawing on one’s professional judgment (Shulman, 1998) to make a steady stream of instructional decisions in the face of consistent pedagogical dilemmas (Lampert, 1985) (see Fig. 1).

By prioritizing pedagogical reasoning as core to teaching practice, our perspective on practice is similar to Sawyer’s (2004) vision of “disciplined improvisation.” Such perspectives assume that teachers consistently face novel problems within dynamic contexts, and that their expertise stems from their ability to engage with those problems as they arise in ways that are contextually appropriate, ethically considered, humane, nurturing, and consistent with disciplinary inquiry. In this way, expertise in teaching is not what Hatano and Inagaki (1986) have called “routine expertise,” meaning the ability to enact difficult routines with predictability and precision. Instead, teaching requires what these same authors call “adaptive expertise,” or the ability to efficiently innovate in the face of novel problems. That said, we acknowledge that teaching involves some routine activity. Männikkö and Husu (2019), along with others (e.g., Anthony, Hunter, & Hunter, 2015; Bransford, Derry, Berliner, Hammerness, & Beckett, 2005; Schwartz, Bransford, & Sears, 2005), have illustrated that teachers’ ability to be adaptive rests on their familiarity with routine instructional practices.

Our belief that ambitious teaching practice is built atop a foundation of pedagogical reasoning has implications for how we prepare novices for the work of teaching. If a TE’s goal is to prepare novice teachers to enact routine instructional moves with precision and predictability, the way she structures practice opportunities will be quite different than if her goal is to prepare novices to use pedagogical reasoning to consider, decide on, and then enact activity in the face of novel pedagogical dilemmas. Understanding this difference was the central project of this research project: within representations, decompositions, and approximations of practice, we wanted to understand what characteristics of teacher education pedagogy granted novice teachers the opportunity to consciously practice engaging in pedagogical reasoning as a central part of preparing for practice.

## 2 Research design and methods

### 2.1 Research participants and context

This study, which emerged from a larger project examining the practice of seven TEs, is a comparative case study of the practice of two TEs, Jenna and Leigh. In both projects, we were interested in understanding if and how participating TEs mediated novice teachers’ pedagogical reasoning when representing, decomposing, and approximating practice. Due to the exploratory nature of this study, we adopted a qualitative methodological approach, which allowed us to follow relevant lines of inquiry as they emerged (Wolcott, 1990).

The TE participants in our larger study were seven content methods instructors (one secondary English language arts, one secondary science, one elementary mathematics, one elementary literacy, and three TEs who were co-teaching the secondary mathematics course). Author A was a participant in the larger study (the ELA methods instructor), but she was not one of the participants in the comparative case study on the practice of Jenna and Leigh that we present here. The other two authors of this article were not participants. All seven TEs were teaching in the same teacher education program, a program that was in its pilot year in the summer of 2014 when data were collected. The program claimed an adaptation of Grossman, Compton, et al.’s (2009) framework as its organizing pedagogical frame for all methods courses (McDonald et al., 2013). Due to this shared framework and to the fact that most TEs had been involved in collaboratively designing the program the previous year around that framework, the program had an unusual amount of pedagogical coherency.

Each TE used a set of instructional activities (IAs) (Lampert & Graziani, 2009) for teaching novices about instructional methods in the content areas, and the TEs heavily relied on representations, decompositions, and approximations to engage novices in learning in and through these IAs. Because all methods instructors used this framework to organize their instruction, this program was a particularly useful site for investigating if and how TEs worked with novice teachers on developing their ability to engage in pedagogical reasoning when representing, decomposing, and approximating practice.

All participants had at one point been classroom teachers in the content area they were teaching, with teaching experience from less than 2 years to more than 10 years. The two TEs we examine as cases in this study were Leigh, a secondary mathematics methods instructor, and Jenna, a secondary science methods instructor (neither are authors of this paper). At the time of data collection, Jenna had 1 year of experience as a TE and Leigh had more than 10 years of experience. Jenna was in her 20s. Leigh was in her 40s. Both were white women. Jenna was a doctoral student of science education whose limited experience teaching science methods coursework was all at one university-based teacher education program. Leigh was a clinical faculty member at a large research university and had taught secondary and elementary mathematics methods courses at a variety of universities and programs. Both Leigh and Jenna had played central roles in designing the practice-based program in which they were teaching.

### 2.2 Data sources

In the larger study, our primary data were 52.5 h of video of methods coursework taught by participating TEs. For this comparative case study, we draw on the 20.45 h of teaching we collected on our case TEs. The video that was captured was determined in advance by the research team, who selected the segments of methods coursework to capture on video based on when TEs were working on focal instructional activities. Each TE identified one focal instructional activity, and video was captured of methods instruction when that instructional activity was being represented, decomposed, and approximated. The goal was to collect only data related to these three teacher education pedagogies. Supplemental data include pre- and post-semi-structured interviews with our two focal TEs and documents related to the methods course (e.g., lesson plans, handouts, and notes).

### 2.3 Data analysis

Our analysis of video data started with our research question: *When working with novice teachers on practice, to what extent and in what ways did TEs mediate novices’ opportunities to engage in pedagogical reasoning?* Guided by this question, our data analysis occurred in three stages. We first ensured that our data included only episodes when practice was represented, decomposed, or approximated by cleaning our data of any moments that were mistakenly collected and did not include any of these three teacher education pedagogies. We then identified episodes in the dataset where TEs were mediating novices’ opportunities to engage in pedagogical reasoning (to understand how we identified episodes, read the “Identifying episodes” section below). We then identified trends within these episodes. We describe this process in the “Identifying trends” section. All video analysis was conducted with Studiocode, video analysis software that allowed us analyze patterns within and across video files. Author A, as both a participant in the larger study and a researcher, participated in the development of the coding scheme; however, she did not code any of the data that were directly related to her methods teaching to ward against biases inherent in self-study (Hamilton, 1998).

#### 2.3.1 Identifying episodes

Because we began with a large data set, our first task was to sample the data that were relevant to our questions. Given our research question, we began with video data and coded the footage that captured moments when practice was being represented, decomposed, or approximated. We excluded any video data that were not coded with one or more of these codes. We divided the remaining data into episodes using a strategy adapted from Horne (2007). Some episodes were single-turn utterances (e.g., “Did you see how I just pressed for justification there?”); others spanned many turns of talk and many participants. Our decision rules for when to end an episode were primarily topical. When talk between TEs and teachers moved away from one teaching decision or action to focus on another, we ended the episode and began another.

#### 2.3.2 Attaching supplemental data to episodes

After the episodes were identified, we drew from lesson plans, interview data, and documents to round out each episode with pertinent data from other sources. To be attached to an episode, supplemental data had to directly pertain to it. For example, after we identified the launch of a particular rehearsal as a pertinent episode and sampled it, we included the following items in the collection of data about the episode: the video footage of the rehearsal launch, the TE’s plan that pertained to that rehearsal launch, the handouts used during that rehearsal launch, and the excerpts from the TE’s interviews when she spoke about the launch.

#### 2.3.3 Coding episodes

To determine whether and how TEs were mediating novices’ opportunities to engage in pedagogical reasoning, we began by using Horne’s (2007) concept of an episode of pedagogical reasoning (EPR), which she defined as “units of teacher-to-teacher talk in which teachers exhibit their understanding of an issue in their practice. Specifically, EPRs are moments in teachers’ interaction in which they describe issues in or raise questions about teaching practice that are accompanied by some elaboration of reasons, explanations, or justifications” (p. 46). Through open coding of the episodes that we identified using Horne’s data analysis strategy, we began to notice patterns, which led us to develop two new units of analysis: the first we called an *episode of pedagogical problem solving* (EPPS) and the second we called an *episode of routine pedagogical practice* (ERPP). We defined EPPSs as units of talk within teacher education coursework in which a problem, issue, or question about pedagogical practice was raised in a way that *did not assume one correct answer or solution*. We defined ERPPs, on the other hand, as units of talk within teacher education coursework in which a specified move, routine, or practice was called on either by a teacher or a TE and discussed in a way that highlighted the importance of executing it *with fidelity to some ideal*. We labeled each episode in our dataset as being either an ERPP, an EPPS, neither, or both. We then looked across the EPPSs to look for trends in teacher education practice. Through iterative review of these episodes, we developed a final codebook with codes drawn from the trends and patterns we identified in the data. These codes included six practices that TEs employed to work on decision-making in practice (for elaborated explanations of these practices, see the discussion section):

We then applied these same codes to the episodes that we had labeled as episodes of routine pedagogical practice (ERPP).

### 2.4 Focal data selection

In the next section, we present and interpret data from two thematically related episodes. In both episodes, the dilemma is “how do I respond to what this student has just said?” One of the episodes was labeled as an ERPP and the other was labeled as an EPPS. In the EPPS sample, the TE explicitly mediates a novice teacher’s attempt to make a deliberate and informed pedagogical decision and employs all of the practices listed in the codes listed in Table 1. In the ERPP sample, when faced with a strikingly similar pedagogical dilemma, the TE employs none of these practices. We offer these contrasting episodes to illustrate how the six practices that emerged from our analytic coding supported the TEs in engaging novices in the complex work of pedagogical reasoning. Neither of the episodes were typical in our data. Both were significantly longer and more explicit. They were selected because their length and depth allow readers to understand how the six practices enumerated above worked together to support TEs to engage novices in deliberate pedagogical decision-making.

**Table 1:** Teacher educator practice codes.

Practice-based teacher educator practices
Creating a shared instructional context
Posing pedagogical dilemmas
Highlighting instructional purposes
Engaging relevant knowledges
Considering multiple instructional decisions
Making instructional decision-making explicit

## 3 Findings

Our analysis revealed that, when representing, decomposing, and facilitating approximations of practice, TEs differed in the extent to which they supported novices to see and practice the pedagogical reasoning that undergirds what Kennedy (2016) would call the “visible behaviors” of teaching. When participating TEs *did* prioritize pedagogical reasoning within their practice-based pedagogy, they employed the practices we highlight in Table 1. We unpack these practices in depth in the “Practice-Based Pedagogy for Pedagogical Reasoning” section later in this paper. However, *first* we offer two contrasting episodes from our data in the hopes of offering readers concrete, extended examples of practice-based pedagogy: one example prioritizes pedagogical reasoning and the other deprioritizes pedagogical reasoning. In our first episode, the TE offers novices a significant amount of support and guidance as she unpacks the pedagogical reasoning that undergirds the practice of eliciting and responding to students.

### 3.1 Supporting novices to engage in pedagogical reasoning

Leigh and Beth co-teach their secondary mathematics methods course. On the third day of their course, they are preparing novice teachers to facilitate a mathematical discussion with high school students. In this discussion, students are meant to match different representations, one graphical and one narrative, of the same mathematical object and discuss their reasoning for matching. To introduce novices to this type of discussion and to the mathematics inside it, Leigh and Beth model a lesson. After modeling the lesson with novices in the student role, Leigh engages in a second model of the same lesson. This time, she asks her novices to collaborate with her as she engages in pedagogical reasoning about what to do in response to a student’s comment.

**Fig. 2:**
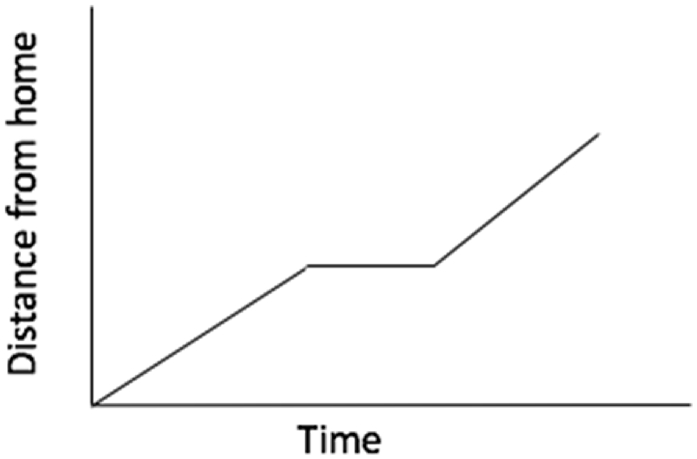
Graph on the board of Leigh and Beth’s classroom.

*As Leigh teaches, on the board are two large pieces of butcher paper. One of the pages reads*: Tom walked slowly along the road, stopped to look at his watch, realized he was late, and then started running. On the other page is this graph (see Fig. 2):

Leigh: I’m going to be the teacher and Beth [*Leigh’s co-teacher*] is going to offer something up and I’m gonna pause us and I’m gonna have us think aloud about what we would do with Beth’s contribution to work towards my [instructional] goal for everyone in the room, okay?.. “Beth, could you explain how you decided that those two [*she points to the story and the graph*] were a match – that that story represented that graph?”

Beth: Well, I saw that it was increasing because he was walking away from home, but then he stopped and looked at his watch, so he wasn’t moving anywhere.

Leigh: … [*To the novice teachers*] In your groups, discuss: what could you do with this [*Beth’s comment*]? What are your options? Come up with a bunch of options of things you could do to pull out the big ideas of what she just said. Go for it, try to be specific, like, what specific questions or moves?”

Here, Leigh has engineered a pedagogical dilemma for her novice teachers by asking Beth to pose a statement she would reasonably expect from students. By asking novices for their next moves, Leigh cues them to the pedagogical dilemma at hand: “what could you do with this [student’s reasoning]?”

*[Novices discuss in their groups. After several minutes, Leigh brings them together again.]* Leigh: All right, what are some of the options that I have as a teacher? What are some of the things I could do?

Novice 1: A follow-up question about what that looks like on the graph. You could say, ‘Could you come up here and point to where that is on the spot?’

Leigh: So a follow-up question. I’m gonna name that a ‘probing’ or ‘clarifying’ question because what you’re doing is you’re probing a little more for specificity. So, you said we could ask a probing or a clarifying question [*she writes ‘probing or clarifying question’ on the board]* … [*Speaking to the whole class]* What I’m trying to do is validate what Beth said and I’m trying to pull out from that the idea that I’m working towards, which is that he wasn’t moving here [*she points to where the line on the graph plateaus*] because he was stopped, right? [*Speaking directly to Novice 1]* So your question serves us – how does it help us work towards that?

Here Leigh returns to and thinks aloud about her two aims as a classroom teacher in this moment: (a) validating student thinking, and (b) building understanding of the graph and what its features represent. By framing the pedagogical dilemma in terms of these instructional and relational purposes, she models one method of decision-making as a teacher.

Novice 1: Well, since she specifically said it’s an increasing graph and then stops, but she didn’t specifically say that’s where the graph gets flat and that’s, like, what your objective is that you’re trying to teach the rest of the class, so maybe some people were following and some maybe not …

Leigh: We want everyone in the room to pick out the key thing in what she said. So, you’re saying, let’s go back to that key thing and ask a probing question. And you actually have a specific *kind* of [probing question], which is what we’re gonna call a linking question [*she writes ‘linking’ on the board]* because you’re asking, “Where in the graph did you see that?” which is linking the story to the graph … I want to get some more ideas on the table first, because there’s a lot of moves we could do and I want to hear some other things we could do in that moment.

By asking this first novice teacher to articulate her ideas with more specificity and then categorizing the kinds of questions the novice teacher brainstorms into types, Leigh brings relevant knowledges to bear on the dilemma while using pedagogies of representation and decomposition.

*Several other novices offer possible responses to Beth’s comment. Similar to the exchange between Leigh and Novice 1, Leigh probes each novice teacher about why they would choose that particular move in light of their instructional goal and other important considerations. For example, after pressing one teacher candidate to offer a rationale for her decision to have another student restate Beth’s comment, Leigh says*, so this is what I was saying earlier about getting specific and getting a rationale—we might ask a probing/clarifying question because one of our goals, or our rationale, is to help connect representations [*she points to both the story problem and the graph*]. Here you’re sort of saying you might open it up to the class because we want to assess if anyone else understands it.

Here, Leigh connects novice teachers’ moves with the instructional purpose behind them. Unsatisfied with simply having many options of teacher moves, she presses novice teachers to discuss *why* they would choose one move—what purpose would each serve, and given her aims, how would this move validate individual student thinking and advance collective understanding for the class?

*[After a handful of novice teachers offered possible responses to Beth’s comment and the class discusses each, Leigh releases novice teachers back into discussion in their small groups.]* Leigh: Could we just take a minute, because we’ve been quiet and talking whole-group for a minute, to think about all of these things *[she points to the list she has written on the board that categorizes possible responses to Beth’s comment]*. Which one feels like the one you would instinctively want to do and which one feels a little bit challenging? [*Novice teachers discuss this question in their table groups. Leigh circulates and listens as groups discuss. After several minutes, Leigh brings the class back together.*]

Leigh: I want to name that this group said that they all had different opinions. And that is awesome, because the whole point is that all of those moves are valid, you just have to be intentional and have a rationale that you can explain. So, does anyone want to say what their rationale was? What they thought they’d do in the moment or what felt comfortable to them? *[As several novices offer how they would respond to Beth’s comment with their accompanying rationale, Leigh records each idea on the board.]*

Again, connecting intentionality and rationale as key considerations in choosing a possible solution, Leigh focuses her representation of pedagogical dilemma management in terms of instructional purpose. Her choice to garner and evaluate many potential solutions from the whole class emphasizes the breadth of possibly effective options.

Leigh: I’m gonna play out what you just did and then I’m gonna wrap it up, so you can go do your rehearsals … We’re gonna work on all the moves you talked about [*she points to the list she created on the board]* – you can use anything you want. [*Pointing to where she has written “*public record” *on the board]* We’re gonna press you on developing a public record that makes really clear what students have said and what your goals are, and we’re gonna push you a little bit on some of these probing or clarifying questions that link representations. So, the public record was things like, moving my finger [*she moves her finger along the graph]* or underlining it [*she uses her finger to mock underline a word in the story problem*] and I’m gonna add in just a second when *I* do this: writing something on the board that summarizes what we said. Um, the probing or linking are things like: “Where did you see him stopped in the graph?” “Where do you see the increasing part on the graph?” So, it’s probing questions that actually force us to connect representations, [*she points to both the story problem and the graph]* which is what we’re doing. *[Following this comment, Leigh jumps back into her model, playing out one possible way of responding to Beth’s comment, and she concludes her model lesson.]*

Leigh’s planning and design clearly frame teaching practice as instructional decision-making. By first planting Beth to offer a typical student response and by facilitating novices in discussing possible responses to Beth’s comment, Leigh engineers an opportunity for her novices to consider an authentic and frequent pedagogical dilemma: in light of my instructional and relational goals, how do I respond to what that student just said? It is through wrestling with this dilemma that Leigh introduces two key teaching moves: creating a public record and asking probing questions. By design, these moves emerge because of their usefulness in solving the authentic pedagogical dilemma Leigh has posed. It is the pedagogical dilemma that offers meaning to and context for working on teaching moves or behaviors in context. Without such situated meaning-making, identifying and working on teaching moves amounts to a drill-and-practice session.

### 3.2 Leaving pedagogical reasoning inexplicit

To contrast with Leigh’s deliberate approach to supporting novices’ pedagogical reasoning practice, we offer another example from our data. This episode offers a strikingly similar dilemma to the one at the center of Leigh’s episode above: “how do I respond to what this student has just shared?” However, this episode was labeled as an “episode of routine pedagogical practice” or ERPP (as opposed to Leigh’s episode, which was labeled as an “episode of pedagogical problem solving” or EPPS). The TE in this next episode does not highlight the pedagogical dilemma on the table, connect the dilemma with an instructional purpose, engage relevant knowledge, consider possible instructional decisions, or make the instructional decision explicit.

Jenna, a secondary science methods instructor, has just introduced a biology instructional activity to her novice science teachers. Their task is to plan questions to accompany students’ viewing of a time-lapse video of an acorn growing into a tree. The questions they have been asked to plan are supposed to elicit student inferences about the unseen factors influencing the tree’s growth. After teachers have planned their question sequence, Jenna leads them in rehearsing asking the questions they have planned.

*John volunteers to act as teacher in the group’s first experience with a formal teaching rehearsal. He starts out with his own planned questions: “What’s happening? What do you notice?” Students respond with three variations on “the plant is getting bigger” and John revoices each one in turn. Then the fourth student explains that “the plant grew towards the light.”*

John: OK, so you’re seeing light in the video. What do you think is causing the growth?

Novice playing a student (1): I remember hearing something about photosynthesis when I learned about this last year.

John: Can you tell me more about photosynthesis?

*[The novice playing a student adds that photosynthesis means converting light and nutrients into energy]*

Novice playing a student (2): I know photosynthesis happens on each cell.

John: [*looks surprised and turns to Jenna, his TE]* Can I ask, “How do you know that?” Or [pause]. How do you respond?

Jenna: You can ask, like, “What do you mean? What do you mean happens at each cell?”

John: [*turns back to Novice 2*] What do you mean? Can you tell me more about that? What do you mean, it happens at each cell?

Here, John has posed an authentic dilemma: how do I respond to this student’s thinking? In this case, instead of keeping the problem space open, Jenna closes it by making the instructional decision herself and feeding John a line. There are many reasons Jenna may have chosen to do this. One possible reason Jenna may have for closing the problem space is that this particular dilemma may not be the most important thing for her novices to be focusing on at this particular moment. Or Jenna may be intentionally treating the work of probing for more information as a routine action that does not require thought. Jenna may feel that teachers should routinely probe for more information and that they do not need to reason through this decision. Lastly, Jenna may have closed the problem space because she simply does not have the time to engage her novices in pedagogical reasoning at the moment. Whatever was motivating her decision, Jenna chose not to mediate the novice teachers’ process of pedagogical reasoning at this moment.

As this rehearsal comes to a close, Jenna again offers John directive feedback rather than mediating his decision-making:

Jenna: When you have students who are responding, it becomes this really intellectual game of how do I respond and what ideas do I pick up? You can see that the content really matters in terms of what you elevate and what you push on.

Jenna closes the rehearsal by naming the dilemma that she saw arising again and again during John’s rehearsal: “how do I respond?” While she sees this as an “intellectual game,” she does not make visible how the game is played.

### 3.3 Practice-Based Pedagogy for Pedagogical Reasoning

We offer these two thematically related episodes from our data to illustrate an example and a non-example of TEs’ work of unpacking the process of engaging in pedagogical reasoning. Both episodes include examples of what our participants and others would describe as practice-based teacher education (PBTE). Both TEs represented and decomposed teaching and supported novices in their own approximations of teaching. Leigh included the invisible process of pedagogical reasoning as a part of the practice that she represented, decomposed, and approximated. Jenna, on the other hand, included only what Kennedy (2016) would call the “visible behaviors” of teaching. She did not explicitly engage novices in practicing the more invisible part of teaching: how to use one’s judgment to make informed instructional choices. We believe that this aspect of a TE’s pedagogy when enacting PBTE may be a significant determinant in whether teaching is treated as a technocratic activity or a complex relational activity requiring specialized professional judgment.

Across our data, when TEs mediated novices’ attempts to engage in pedagogical reasoning, they employed a collection of practices that were woven throughout their representations, decompositions, and approximations of practice. While all TEs represented, decomposed, and approximated practice, these more nuanced practices distinguished one approximation from another, one representation from another, or one decomposition from another. We refer to these practices as *textural* because they were woven throughout whatever a TE was doing. These practices emerged from our data and became the final codes that we applied during analysis (Table 1). We distinguish these textural practices from the more *temporal* practices (practices that had beginnings, middles, and ends), such as representing teaching by screening video, or approximating teaching by facilitating a rehearsal. While the TEs all employed the same temporal practices (all screened video, all modeled teaching, all facilitated rehearsals), the texture of their practice within those activities differed greatly.^1^ In the next paragraphs, we unpack each of the textural practices outlined in Table 1 and discuss how they showed up in our data.

Across our data, whether an episode was labeled as an *episode of pedagogical problem solving* (EPPS) or an *episode of routine pedagogical practice* (ERPP), when TEs worked on practice, they first **created a shared instructional context** within which to unpack the nuances of teaching practice. In Leigh’s episode, a shared instructional context was created through her model lesson, whereas in Jenna’s episode, the shared instructional context was John’s rehearsal. In other episodes in our data, TEs created shared instructional contexts by screening a video, discussing a written transcript of classroom talk, discussing a lesson plan, or engaging novices in a reflection on their individual teaching of a common lesson. Of the six practices that we identified in our analysis, creating shared instructional context was the only practice that consistently showed up across our dataset in both EPPSs and ERPPs. It is interesting to note that this practice is significantly more temporally bound than the others. TEs usually create a shared instructional context by engaging novices in an activity that has a beginning, middle, and end. Looking across our data, the other five practices that we identified emerged almost exclusively in the episodes in our data coded as EPPSs and were much more textural—in other words, they wove in and out of many different kinds of teacher learning activities and didn’t have neat beginnings, middles, and ends.

Across all identified EPPSs in our data, TEs **posed pedagogical dilemmas**: forced choices between equally reasonable instructional alternatives (Lampert, 1985). The most common pedagogical dilemma was some version of “What should I do/say next?” In Leigh’s episode, the dilemma was, “how should I respond to Beth’s idea?” While there was a pedagogical dilemma in Jenna’s episode, it was posed by John, a novice, and not by Jenna (“how do I respond to this student?”). Because Jenna did not engage John’s dilemma explicitly as a decision-making process involving judgment, we did not apply this code to her practice. Across our data, TEs posed pedagogical dilemmas in a variety of ways: they stopped the action of a video, model, or rehearsal, or they pointed to particular moments within written transcripts or lesson plans and raised questions about what a teacher might do next and why.

In those episodes coded as EPPSs, TEs also **highlighted instructional purposes**. These purposes became the anchors around which they supported novices to practice making principled instructional decisions. While Jenna did not do this in her episode, Leigh’s episode offered multiple examples. She highlighted an instructional purpose every time she described a goal (e.g., “what I’m trying to do is validate what Beth said,” “we might ask a probing/clarifying question *because one of our goals, or our rationale, is to help connect representations”*). Examples from other episodes in our dataset include, “If you were the teacher and your goal was *to get more students involved in the discussion*, what would you do next and why?” or “Let’s stop here for a second—we’ve veered away from your instructional goal. If *we want to focus ourselves back towards our instructional goal*, what could we do at this point?” Common instructional purposes in our dataset were: eliciting students’ ideas; engaging more students in learning; redirecting student behaviors that are not supporting a productive learning environment; explaining content; assessing student understanding; and orienting students to each other’s ideas.

Along with posing pedagogical dilemmas and highlighting instructional purposes, in the EPPSs across our dataset, TEs also **considered multiple instructional decisions**. While Jenna offered John only one teaching move as an option, Leigh engaged novices in brainstorming many options for how to respond to Beth. She was also explicit about why she had novices consider multiple options, “I want to name that this group said that they all had different opinions. And that is awesome, because the whole point is that all of these moves are valid, you just have to be intentional and have a rationale that you can explain.” When only one answer is presented as a way to address a dilemma, it can seem like there is ‘one right way’ to teach. By engaging novices in considering multiple options and explicitly highlighting that multiple routes may be appropriate, Leigh frames teaching as a complex activity that requires contextual pedagogical reasoning rather than context-independent “best practices.”

While considering these multiple options, TEs in the data set explicitly taught novices by modeling and offering guidance in how to **engage relevant knowledges**. The vast professional knowledge base of teaching (Wilson, Shulman, & Richert, 1987) demands much of teachers: knowledge of subject matter, pedagogical content, general pedagogy, other content, curriculum, learners, social structures and how they impact the classroom space, and educational aims—and they must also possess and access structural understanding of these knowledges in order to choose and create representations of content that will prove effective for particular learners in a particular context. TEs supported novices in developing and engaging this professional knowledge base in many ways: by uncovering several possible ways to address the dilemma and, in discussing the affordances and constraints of each possibility, bringing forth knowledge about students, content, informational tools and supports, and teaching and learning.

Finally, TEs **made instructional decisions explicit** rather than enacting specified moves (or supporting novices to enact moves) without highlighting the pedagogical reasoning that undergirded them. One such moment in Leigh’s episode stands out. After the class had considered many possible moves and discussed rationale, affordances, and constraints of each, Leigh said,

I’m gonna play out what you just [decided] … We’re gonna work on all the moves you talked about … and I’m gonna add in just a second when *I* do this: writing something on the board that summarizes what we said. Um, the probing or linking are things like: “Where did you see him stopped in the graph?” “Where do you see the increasing part on the graph?” So, it’s probing questions that actually force us to connect representations, [*she points to both the story problem and the graph]* which is what we’re doing. *[Following this comment, Leigh jumps back into her model, playing out one possible way of responding to Beth’s comment, and then she concludes her model lesson.]*

Here, rather than keeping her pedagogical reasoning to herself, Leigh made her final decision explicit by naming and describing it. Her decision-making about the dilemma was both collaborative and guided. By orienting to the decision-making process—rather than to fidelity to a specific method or strategy—she provides an opportunity for novices to practice pedagogical reasoning (Kavanagh, Metz et al., 2019). Looking back to Fig. 1, we can see that in the hands of a sophisticated TE, making instructional decisions explicit is not an end goal but merely a stop along on ongoing cycle. Every decision a teacher makes inevitably leads to more dilemmas (Lampert, 1985).

## 4 Implications and conclusions

We embarked on this research because we take seriously a set of critical questions that scholars have raised about PBTE: Can teacher education that is grounded in predetermined, pre-specified routines and practices support novices in learning to enact teaching that is responsive to students and to context (Kennedy, 2016; Philip et al., 2019; Zeichner, 2012)? Can specifying core practices (Grossman, Dean, Kavanagh, & Herrmann, 2019; Grossman, Hammerness, & McDonald, 2009), routine instructional activities (Lampert & Graziani, 2009), and teacher education pedagogies (Grossman, Kavanagh, & Dean, 2018, McDonald, Kazemi, & Kavanagh, 2013) support the development of teaching expertise that is more adaptive than routine (Hatano & Inagaki, 1986)? Spurred by these questions, we set out to investigate whether and to what extent TEs in a practice-based program created opportunities for novices to practice engaging in pedagogical reasoning. Did TEs engaged in practice-based pedagogy simply work on technique or did they also offer practical scaffolds for novices to develop and use their emergent professional judgment through opportunities to engage in pedagogical reasoning?

By focusing on the episodes of methods instruction within our dataset where novices’ instructional decision-making was highly mediated, our analysis revealed a collection of practices that, when employed in various combinations, seemed to support novices in practicing the invisible work of identifying dilemmas, using judgment tied to disciplinary goals and professional ethics to consider alternatives, and making informed instructional decisions. We view this approach to PBTE as aligned with a vision of expertise in teaching as adaptive and not routine (Hatano & Inagaki, 1986). As more programs adopt practice-based approaches to teacher education, it will be important to continue enriching the field’s knowledge about how to design PBTE instruction that supports the development of adaptive expertise and specialized professional judgment.

The findings of this study indicate that when representing, decomposing, and approximating teaching, TEs can support novices in ways that align with visions of teaching that value professional judgment and responsiveness to context. This is significant because prominent scholars have begun to question whether the grounding assumptions of PBTE make this type of work untenable (Kennedy, 2016; Philip et al., 2019; Zeichner, 2012). Philip et al. (2019) have claimed that “calls for reductive notions of PBTE defined by routines and positioned in contrast to theory have grown in popularity” in recent years, and they go on to argue that PBTE represents “packaged, ready-to-use models of teacher training” (p. 2). Additionally, Zeichner (2012) and Kennedy (2016) have expressed concerns about the role of judgment in PBTE. We believe that the findings of this study demonstrate that practice-based teacher education pedagogies can be in alignment with perspectives on teaching that value pedagogical reasoning. Therefore, this study’s findings contribute to ongoing work to justify and improve PBTE, especially when taken together with other recent studies (Dutro & Cartun, 2016; Kavanagh & Danielson, 2019; Kavanagh, Metz et al., 2019; Kavanagh, Monte-Sano et al., 2019; Kavanagh & Rainey, 2017; Reisman et al., 2019).

Second, our findings offer teacher education researchers an additional lens for examining the pedagogy of teacher education. The lexicon for describing the pedagogical practice of TEs remains thin, and the language describes the component parts of TEs’ practice at a very large grain (Grossman, Compton, et al., 2009). By offering language to describe the pedagogical practice of TEs at a finer grain than words like *inquiry, reflection, representation, decomposition,* and *approximation,* researchers may begin to investigate the pedagogy of TEs with more nuance.

Finally, we turn to the practical implications of our study. While the data that we analyzed for this study were from content-based instructional methods coursework and not foundations coursework, a TE teaching a practice-based foundations class focused on teaching for educational justice could employ the same textural practices to support novices in engaging in pedagogical reasoning informed by knowledge about historical and ongoing structures of oppression, or students’ cultural funds of knowledge and linguistic resources in their teaching context. It is not difficult to imagine an episode similar to Leigh’s, where a TE plants a student comment about race, gender, or sexuality and then engages novices in discussing the affordances and constraints of a variety of responses calling on knowledge about privilege and power and how they operate in classroom spaces. While practice-based approaches to teaching about justice and equity are not widespread (for exceptions see Dutro & Cartun, 2016; Kavanagh, 2017; Kavanagh & Danielson, 2019), MacPherson (2010) has offered the field a conceptualization of intercultural teaching as decision-making; this work, together with one or more of the many comprehensive models of social justice teaching (e.g., Lynch and Baker’s (2005) “equality of condition” framework, Kumashiro’s (2002) “anti-oppressive education” framework, Banks’s (1995) “five dimensions of multicultural education” framework, Paris’s (2012) “culturally sustaining pedagogy” framework, or North’s (2006) “three spheres of social justice” framework) could be instructive for further examining the implications of the findings of this study for teacher education in the foundations (Kavanagh & Danielson, 2019).

A second implication of our findings has to do with how we, as a field, support teacher educators’ to develop their practice. Various organizations have begun to design and implement professional development for TEs focused on supporting TEs’ pedagogical practice. While, we believe that sophisticated practice in teacher education can be taught and learned and that the field would benefit from high-quality professional development for TEs, given the paucity of research on the practice of TEs, efforts to prepare TEs are frequently not grounded in research. This study, together with our related research (Kavanagh & Danielson, 2019; Kavanagh, Metz et al., 2019; Kavanagh, Monte-Sano et al., 2019; Kavanagh & Rainey, 2017), might inform professional development efforts aimed at TEs.

Practice-based tools (core practices, pedagogies of practice) are widely used in teacher education. While these tools are promising supports for TEs, they can be used to promote complex pedagogical reasoning skills or to instantiate rote teaching. The field has few representations of what it looks like to use these tools in the service of visions of ambitious teaching. With this study, we offer a more nuanced vision of PBTE in the service of teaching that values professional judgment and adaptive expertise. By developing a vision of high-quality PBTE, we hope to support the field in better aligning teacher education pedagogy and instructional design with ambitious teaching practice.

## Notes

The authors would like to acknowledge the contributions of Emily Shahan, Andrea Bien, Megan Kelley-Petersen, Katie Danielson, Annie Garrison Wilhelm, Morva McDonald, Hannah Neiman, Dawn Woods, and Kara Jackson.

This work has been supported by the Bill & Melinda Gates Foundation [grant number OPP1071939]. Any opinions, findings, and conclusions or recommendations expressed in this material are those of the authors and do not necessarily reflect the views of the funders.

## CRediT authorship contribution statement

**Sarah Schneider Kavanagh:** Conceptualization, Methodology, Formal analysis, Investigation, Resources, Data curation, Writing - original draft, Writing - review & editing, Supervision, Project administration. **Jenni Conrad:** Writing - review & editing. **Sarah Dagogo-Jack:** Formal analysis.

## Footnotes

1 The distinction between “temporal” and “textual” practices may be a useful heuristic for analyzing *teaching* practices, as well as for analyzing *teacher education* practices. For example, while “facilitating discussion” is a temporal practice (it has a beginning, a middle, and an end), “positioning students as competent sense-makers” and “orienting students to each other’s ideas” (Kazemi & Cunard, 2016; Kavanagh & Rainey, 2017; Reisman et al., 2018) are *textural* practices – they weave in and out of *many* temporally bounded practices, including facilitating discussion.

## References

[bib1] Anthony G., Hunter J., Hunter R. (2015). Prospective teachers development of adaptive expertise. Teaching and Teacher Education.

[bib2] Banks J.A. (1995). Multicultural education and curriculum transformation. The Journal of Negro Education.

[bib3] Bird T., Little J.W. (1986). How schools organize the teaching occupation. The Elementary School Journal.

[bib4] Boerst T., Sleep L., Ball D., Bass H. (2011). Preparing teachers to lead mathematics discussions. Teachers College Record.

[bib5] Brandenburg R., Glasswell K., Jones M., Ryan J. (2017). Reflective theories in teacher education practice: Process, impact, and enactment.

[bib6] Bransford J., Derry S., Berliner D.C., Hammerness K., Beckett K.L., Darling-Hammond L., Bransford J. (2005). Theories of learning and their roles in teaching. Preparing teachers for a changing world.

[bib7] Carr W., Kemmis S. (1986). Becoming critical: Education, knowledge, and action research.

[bib8] Chazan D., Herbst P.G., Clark L.M., Gitomer D.H., Bell C.A. (2016). Research on the teaching of mathematics: A call to theorize the role of society and schooling in mathematics instruction. Handbook of research on teaching.

[bib9] Cochran-Smith M., Cannady M., McEachern K.P., Viesca K., Piazza P., Power C. (2012). Teachers’ education and outcomes: Mapping the research terrain. Teachers College Record.

[bib10] Cochran-Smith M., Lytle S. (1993). Inside/outside teacher research and knowledge.

[bib11] Cochran-Smith M., Lytle S. (2009). Inquiry as stance: Practitioner research for the next generation.

[bib12] Darling-Hammond L., Bransford J. (2005). Preparing teachers for a changing world: What teachers should learn and be able to do.

[bib13] Dewey J. (2010). Democracy and education.

[bib14] Dutro E., Cartun A. (2016). Cut to the core practices: Toward visceral disruptions of binaries in practice-based teacher education. Teaching and Teacher Education.

[bib15] van Es E., Sherin M.G. (2008). Mathematics teachers “learning to notice” in the context of a video club. Teaching and Teacher Education.

[bib16] Ghousseini H., Herbst P. (2016). Pedagogies of practice and opportunities to learn about classroom mathematics discussions. Journal of Mathematics Teacher Education.

[bib17] Gibbons L.K., Kazemi E., Lewis R.M. (2017). Developing collective capacity to improve mathematics instruction: Coaching as a lever for school-wide improvement. The Journal of Mathematical Behavior.

[bib18] Gore J.M. (1987). Reflecting on reflective teaching. Journal of Teacher Education.

[bib19] Gore J.M., Zeichner K.M. (1991). Action research and reflective teaching in preservice teacher education: A case study from the United States. Teaching and Teacher Education.

[bib20] Grossman P., Compton C., Igra D., Ronfeldt M., Shahan E., Williamson P.W. (2009). Teaching practice: A cross professional perspective. Teachers College Record.

[bib75] Grossman P., Dean C.P., Kavanagh S.S., Herrmann Z. (2019). Preparing teachers for student-centered teaching: The core practices of project-based teaching. Phi Delta Kappan.

[bib21] Grossman P., Hammerness K., McDonald M. (2009). Redefining teaching, re-imagining teacher education. Teachers and Teaching: Theory and Practice.

[bib73] Grossman P., Kavanagh S.S., Dean C.P., Grossman P. (2018). The turn to practice in teacher education. Teaching core practices in teacher education.

[bib70] Grossman P., Kazemi E., Kavanagh S.S., Franke M., Dutro E. (2019). Learning to facilitate discussions: Collaborations in practice-based teacher education. Teaching & Teacher Education.

[bib22] Hamilton M.L. (1998). Reconceptualizing teaching practice: Self-study in teacher education.

[bib23] Hatano G., Inagaki K., Stevenson H., Azuma H., Hakuta K. (1986). Two courses of expertise. Child development and education in Japan.

[bib64] Hauser M., Kavanagh S.S., Nolbit G.W. (2019). Practice-based teacher education. Oxford research encyclopedia of education.

[bib24] Horne I.S. (2007). Fast kids, slow kids, lazy kids: Framing the mismatch problem in mathematics teachers’ conversations. The Journal of the Learning Sciences.

[bib25] Janssen F.J.J.M., Grossman P., Westbroek H.B. (2015). Facilitating decomposition and recomposition in practice-based teacher education: The power of modularity. Teaching and Teacher Education.

[bib74] Kavanagh S., Brandenburg R., Glasswell K., Jones M., Ryan J. (2017). Practicing social justice: Towards a practice based approach to learning to teach for social justice. Reflective theories in teacher education practice: Process, impact, and enactment.

[bib68] Kavanagh S.S., Danielson K. (2019). Practicing justice, justifying practice: Towards critical practice teacher education. American Educational Research Journal.

[bib67] Kavanagh S.S., Metz M., Hauser M., Fogo B., Taylor M., Carlson J. (2019). Practicing responsiveness: Using approximations of teaching to build teachers’ instructional judgment. Journal of Teacher Education.

[bib69] Kavanagh S.S., Monte-Sano C., Reisman A., Fogo B., McGrew S., Cipparone P. (2019). Teaching content in practice: Investigating rehearsals of social studies discussions. Teaching and Teacher Education.

[bib66] Kavanagh S.S., Rainey E. (2017). Learning to support adolescent literacy: Teacher educator pedagogy and novice teacher take up in secondary English language arts teacher preparation. American Educational Research Journal.

[bib26] Kazemi E., Cunard A. (2016). Orienting students to one another and to the mathematics during discussions.

[bib27] Kennedy M. (2016). Parsing the practice of teaching. Journal of Teacher Education.

[bib28] Klette K. (2007). Trends in research on teaching and learning in schools: Didactics meet classroom studies. European Educational Research Journal.

[bib29] Kumashiro K. (2002). Against repetition: Addressing resistance to anti-oppressive change in the practices of learning, teaching, supervising, and researching. Harvard Educational Review.

[bib30] Lampert M. (1985). How do teachers manage to teach? Perspectives on problems in practice. Harvard Educational Review.

[bib31] Lampert M. (2010). Learning teaching in, from, and for practice: What do we mean?. Journal of Teacher Education.

[bib32] Lampert M., Beasley H., Ghousseini H., Kazemi E., Franke M., Stein M.K., Kucan L. (2010). Using designed instructional activities to enable novices to manage ambitious mathematics teaching. Instructional explanations in the disciplines.

[bib33] Lampert M., Franke M.L., Kazemi E., Ghousseini H., Turrou A.C., Beasley H., Crowe K. (2013). Keeping it complex: Using rehearsals to support novice teacher learning of ambitious teaching. Journal of Teacher Education.

[bib34] Lampert M., Graziani F. (2009). Instructional activities as a tool for teachers’ and teacher educators’ learning. The Elementary School Journal.

[bib35] Lemov D., Oct. 26 (2012). Practice makes perfect – and not just for jocks and musicians. https://www.wsj.com/articles/SB10001424052970204530504578078602307104168.

[bib36] Lemov D., Woolway E., Yezzi K. (2012). Practice perfect: 42 rules for getting better at getting better.

[bib37] Loughran J. (2019). Pedagogical reasoning: The foundation of the professional knowledge of teaching. Teachers and Teaching.

[bib38] Lynch K., Baker J. (2005). Equality in education: An equality of condition perspective. School Field.

[bib39] MacPherson S. (2010). Teachers’ collaborative conversations about culture: Negotiating decision making in intercultural teaching. Journal of Teacher Education.

[bib40] Männikkö I., Husu J. (2019). Examining teachers’ adaptive expertise through personal practical theories. Teaching and Teacher Education.

[bib65] McDonald M., Kazemi E., Kavanagh S.S. (2013). Core practices and pedagogies of teacher education: A call for a common language and collective activity. Journal of Teacher Education.

[bib41] Moje E.B. (2015). Doing and teaching disciplinary literacy with adolescent learners: A social and cultural enterprise. Harvard Educational Review.

[bib42] North C.E. (2006). More than words? Delving into the substantive meaning(s) of “social justice” in education. Review of Educational Research.

[bib43] Nystrand M., Wu L.L., Gamoran A., Zeiser S., Long D.A. (2003). Questions in time: Investigating the structure and dynamics of unfolding classroom discourse. Discourse Processes.

[bib44] Paris D. (2012). Culturally sustaining pedagogy: A needed change in stance, terminology, and practice. Educational Researcher.

[bib45] Philip T.M., Souto-Manning M., Anderson L., Horn I.S., Andrews D.C., Stillman J. (2019). Making justice peripheral by constructing practice as ‘core’: How the increasing prominence of core practices challenges teacher education. Journal of Teacher Education.

[bib71] Reisman A., Cipparone P., Jay L., Monte-Sano C., Kavanagh S.S., McGrew S., Fogo B. (2019). Evidence of emergent practice: Teacher candidates facilitating historical discussions in their field placements. Teaching & Teacher Education.

[bib72] Reisman A., Kavanagh S.S., Monte-Sano C., Fogo B., Simmons E., Cipparone P. (2018). Facilitating whole-class discussions in history: A framework for preparing teacher candidates. Journal of Teacher Education.

[bib46] Sawyer R.K. (2004). Creative teaching: Collaborative discussion as disciplined improvisation. Educational Researcher.

[bib47] Schön D.A. (1983). The reflective practitioner: How professionals think in action.

[bib48] Schwartz D.L., Bransford J.D., Sears D., Mestre J.P. (2005). Efficiency and innovation in transfer. Transfer of learning from a modern multidisciplinary perspective.

[bib49] Shanahan T., Shanahan C. (2012). What is disciplinary literacy and why does it matter?. Topics in Language Disorders.

[bib50] Shulman L. (1987). Knowledge and teaching: Foundations of the new reform. Harvard Educational Review.

[bib51] Shulman L.S. (1998). Theory, practice, and the education of professionals. The Elementary School Journal.

[bib52] Sparkes A.C. (1991). The culture of teaching, critical reflection and change: Possibilities and problems. Educational Management Administration & Leadership.

[bib53] Stenberg K., Karlsson L., Pitkäniemi H., Maaranen K. (2014). Beginning student teachers’ teacher identities based on their practical theories. European Journal of Teacher Education.

[bib54] Tiilikainen M., Toom A., Lepola J., Husu J. (2019). Reconstructing choice, reason and disposition in teachers’ practical theories of teaching (PT). Teaching and Teacher Education.

[bib63] Von Esch K., Kavanagh S.S. (2018). Preparing mainstream classroom teachers of English language learner (EL) students: Grounding practice-based designs for teacher learning in theories of expertise development. Journal of Teacher Education.

[bib55] Wetzel M.M., Rosner N.L., Hoffman J.V., Martinez R.A., Price-Dennis D. (2016). “I couldn’t have learned this any other way”: Learning to teach literacy across concurrent practicum experiences. Action in Teacher Education.

[bib56] Wilson S.M., Shulman L.S., Richert A.E., Calderhead J. (1987). ‘150 Different ways’ of knowing: Representations of knowledge in teaching. Exploring teachers’ thinking.

[bib57] Windschitl M., Thompson J., Braaten M., Stroupe D. (2012). Proposing a core set of instructional practices and tools for teachers of science. Science Education.

[bib58] Wineburg S., Martin D., Monte-Sano C. (2013). Reading like a historian: Teaching literacy in middle and high school history classrooms.

[bib59] Wolcott H.F. (1990). Writing up qualitative research.

[bib60] Zeichner K. (2010). Rethinking the connections between campus courses and field experiences in college- and university-based teacher education. Journal of Teacher Education.

[bib61] Zeichner K. (2012). The turn once again toward practice-based teacher education. Journal of Teacher Education.

[bib62] Zierer K. (2015). Educational expertise: The concept of ‘mind frames’ as an integrative model for professionalization in teaching. Oxford Review of Education.

